# Characteristics of Executive Function in School‐Aged Girls With Idiopathic Central Precocious Puberty

**DOI:** 10.1002/brb3.71312

**Published:** 2026-03-31

**Authors:** Qian Zhang, Nai‐jun Wan

**Affiliations:** ^1^ Department of Pediatrics, Beijing Jishuitan Hospital Capital Medical University Beijing China

**Keywords:** executive function, idiopathic central precocious puberty, interference control, internalizing problems

## Abstract

**Aim:**

To investigate the characteristics of executive function (EF) in girls with idiopathic central precocious puberty (ICPP).

**Methods:**

This study included school‐aged girls who were newly diagnosed with ICPP and had not received treatment, along with age‐matched healthy controls. Flanker, Go/No‐Go, and digit span forward and backward tasks were used to evaluate EF. Parents completed the Swanson, Nolan, and Pelham rating scale—IV (SNAP‐IV) and child behavior checklist (CBCL) scales, and family background information was collected. Group differences and associations between task performance and psychological problems were analyzed.

**Results:**

Girls with ICPP showed significantly slower reaction times (RT) in the Flanker task [1.90s (1.34, 2.35) vs. 1.38s (1.19, 1.73), *p* = 0.005], while no significant differences were observed in Flanker accuracy (ACC) or in other EF tasks. On the CBCL, the ICPP group scored significantly higher on internalizing problems [8.0 (6.0, 13.0) vs. 3.5 (3.0, 8.0), *p* = 0.001]. In the ICPP group, Flanker RT was positively correlated with internalizing problem scores (r = 0.766, *p* < 0.001). Multiple regression analysis confirmed that internalizing problems were independent predictors of Flanker RT.

**Conclusion:**

Girls with ICPP demonstrated a deficit in interference control, which was closely associated with internalizing problems.

## Graphical abstract

This study examined executive function (EF) in school‐aged girls with idiopathic central precocious puberty (ICPP). Using computerized cognitive tasks and behavioral scales, we found a selective impairment in interference control rather than generalized EF deficit. This interference control problem was significantly associated with internalizing problems.

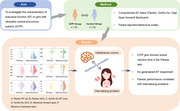

## 1 Introduction

Precocious puberty (PP) is an endocrine disorder characterized by the early development of secondary sexual characteristics and premature maturation of the reproductive organs, which leads to abnormal growth and development in children. The condition is much more common in girls than in boys. Multicenter studies have shown that the prevalence of PP girls has increased from 2.91% to 4.74%, showing a rising trend and posing a serious threat to children's healthy development (Liu et al. 2021, Zhu et al. 2013, Liang et al. 2023). Idiopathic central precocious puberty (ICPP) is one of the most common types of central precocious puberty (CPP), resulting from the premature secretion of gonadotropin‐releasing hormone (GnRH), which activates the hypothalamic‐pituitary‐gonadal axis (HPGA) earlier than normal. Once the HPGA is activated, it is difficult for them to return to a normal development course without appropriate medical intervention. Therefore, the potential consequences of ICPP are generally more severe than those of peripheral precocious puberty (PPP).

Previous studies have confirmed that ICPP can impair children's growth potential and negatively affect their psychological health (Zevin and Eugster 2023, López‐Miralles et al. 2022). Building on this foundation, cognitive and neuropsychological research suggests that ICPP may disrupt critical stages of brain development. The premature exposure to sex hormones is hypothesized to interfere with the maturation of neural networks involved in cognition and emotional regulation, especially executive function (EF) (Qin et al. 2025). These alterations may interfere with their daily life and academic performance, further worsening psychological problems (Suutela et al. 2022). These findings indicate that the impact of ICPP on children may exceed our previous understanding. Since EF reaches a critical stage of neurocognitive development during puberty, an in‐depth investigation of EF in children with ICPP is necessary.

## 2 Methods

### 2.1 Study Design and Participants

This study adopted a cross‐sectional design. We recruited school‐aged girls between 6 and 12 years old who were newly diagnosed with ICPP and had not received any treatment, and age‐matched healthy control girls. All participants were enrolled in mainstream schools in mainland China, rather than special education schools or specialized classes. Participants were recruited between December 2023 and December 2024 from the Department of Pediatric Endocrinology and the Child Health Clinic of Beijing Jishuitan Hospital, Capital Medical University.

Inclusion criteria: ICPP group: (1) Girls children aged 6–12 years; (2) meeting the diagnostic criteria for ICPP as defined in *Expert* Consensus on the Diagnosis and Treatment of Central Precocious Puberty (2022) (Society of Pediatric Endocrinology, Metabolism, and Genetics, Chinese Society of Pediatrics 2023); (3) complete clinical data and good compliance; (4) parents or legal guardians agreed to participate in the interviews and assessments and signed informed consent. Healthy control group: (1) Healthy girls aged 6–12 years with Tanner stage I and no clinical signs of puberty onset; (2) girls aged 7.5–12 years whose Tanner stage was consistent with their age.

Exclusion criteria: (1) Secondary, peripheral, or contrasexual precocious puberty; (2) presence of severe infections, malignancies, cardiac, pulmonary, hepatic, or renal dysfunction, hematologic disorders, or severe psychiatric diseases; (3) chromosomal abnormalities; (4) other endocrine, genetic, metabolic, or central nervous system disorders.

Ethical approval for this study was obtained from the Beijing Jishuitan Hospital, Capital Medical University, Beijing, China (approval number: K2024‐195‐00). All data collection was conducted with written informed consent from parents or legal guardians. Additionally, for children aged 8 years or older, assent from the child was also obtained. All data were handled in compliance with applicable data protection regulations, and confidentiality was strictly maintained throughout the study. This study complies with the ethical principles outlined in the Declaration of Helsinki.

### 2.2 Assessment of EF

This study focused on two core dimensions of EF: inhibitory control and working memory, which play essential roles in children's cognitive development. To ensure comprehensive and stable evaluation of EF, two tasks were selected for each core dimension. The combination of different task modalities are response requirement allowed for control of task‐specific biases and improved generalizability of assessment.

The assessment program was conducted by a software application entitled Brain Function Information Management Platform Software System (product model: CCRT‐C), which was developed by Beijing Wispirit Technology Co., Ltd.

#### 2.2.1 Inhibitory Control Assessment

##### 2.2.1.1 Flanker Task

In this task, a row of five arrows was presented on the screen, with the middle arrow serving as the target stimulus. Participants were instructed to press a key corresponding to the direction of the central arrow: the left key for left‐pointing arrows (←) or the right key for right‐point (→).

**FIGURE 1:**
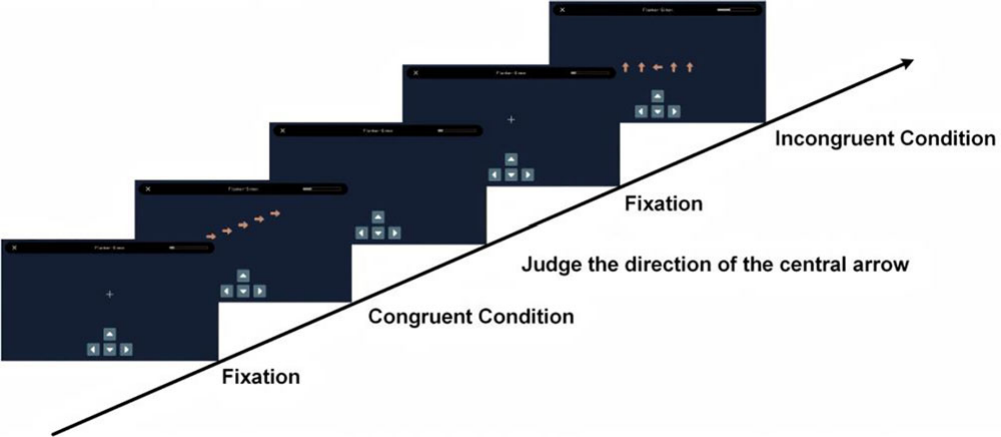
Structure of the flanker task. Participants responded to the direction of the central arrow while ignoring the Flankers. Trials included congruent (e.g., →→→→→) and incongruent (e.g., ↑→←↓→).

The task had a total of 36 formal trials, including 18 congruent trials (all arrows pointed in the same direction as the target, e.g., ←←←←← or →→→→→) and 18 incongruent trials (the arrows pointed in the opposite direction, e.g., ↑→←↓→, or ←←→←←). The order of the trials was randomized. The detailed procedure is shown in Figure 1.

Each trial was presented with a response window of 3300 ms. If no response was made within this time window, the trial was recorded as incorrect, and the trial duration was recorded as 3300 ms. Each trial was followed immediately by the next stimulus. The total task duration was up to 120 s. Reaction time (RT) and accuracy (ACC) were recorded for each trial.

##### 2.2.1.2 Go/ No‐Go Task

**FIGURE 2:**
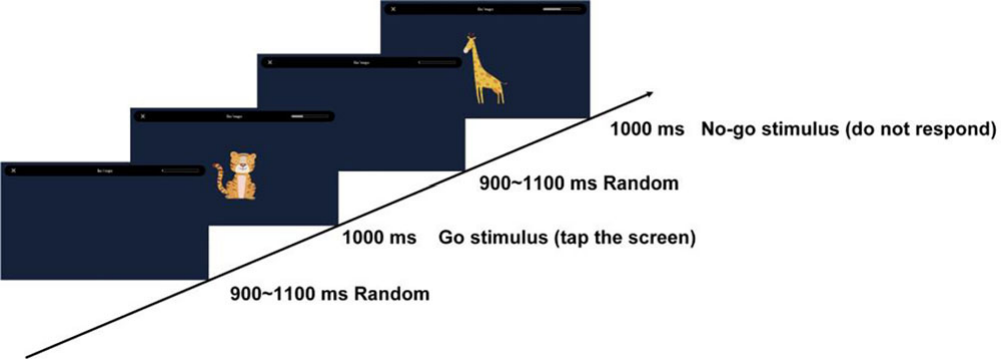
Structure of the Go/No‐Go task. Participants pressed a key when a tiger/lion image appeared (Go trials) and withheld responses to giraffe/elephant images (No‐Go trials).

In this task, participants were shown pictures of animals (tiger and giraffe in the first round; lion and elephant in the second round). Each picture was displayed for 1000 ms, followed by a blank screen with a variable interstimulus interval (ISI) of 900–1100 ms. Participants were instructed to press a key as quickly as possible when a Go stimulus (tiger or lion) appeared and to withhold responses when a No‐Go stimulus (giraffe or elephant) appeared (Figure 2).

Each round contained 100 trials with a 1:1 ratio of Go to No‐Go stimuli presented in random order. RT for correct Go trials (ms) and ACC (correct Go + correct No‐go/total) were recorded.

#### 2.2.2 Working Memory Assessment

##### 2.2.2.1 Digit Span Forward

**FIGURE 3:**
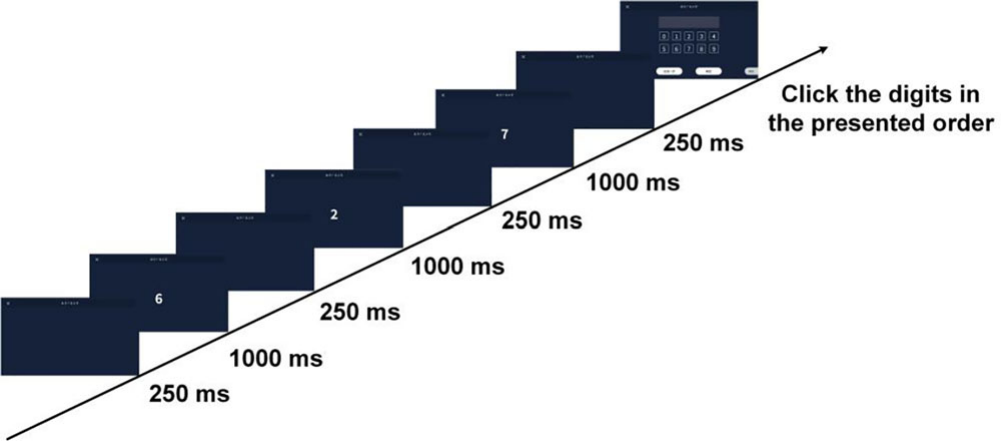
Structure of the digit span forward task. Digits were sequentially presented on a screen, and participants were required to recall them in the same order. Sequence length increased progressively until task termination.

Numbers were presented one by one in the center of the screen, each for 1000 ms with a 250 ms ISI between digits. After the sequence ended, participants were asked to repeat the digits in the same order as presented (e.g., for “6–2–7”, the correct response was “6–2–7”). The detailed procedure is shown in Figure 3.

The task started with a sequence length of two digits, increasing by one digit after each successful trial, up to a maximum of 15 digits or until two consecutive errors occurred at the same length. The maximum forward span was recorded. This task primarily assessed short‐term storage and sequential recall in visual working memory.

##### 2.2.2.2 Digit Span Backward

**FIGURE 4:**
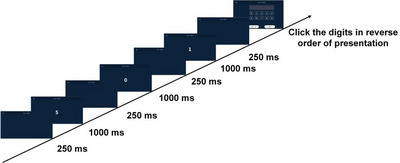
Structure of the digit span backward task. Digits were sequentially presented on a screen, and participants were required to recall them in reverse order. Sequence length increased progressively until task termination.

The presentation of the digit sequence was identical to the Forward task. However, on completion of the sequence, participants were required to repeat the digits in reverse order (e.g., for “5–0–1”, the correct response was “1–0–5”). The detailed procedure is shown in Figure 4.

The sequence length began at two digits and increased by one after each successful trial, up to 15 digits or until two consecutive errors occurred. The maximum backward span was recorded. This task assessed the manipulation and updating components of visual working memory.

### 2.3 Parent Interviews and Scales Assessments

To supplement the child performance data, primary caregivers were interviewed and asked to complete standardized scales and a family survey. These include: (1) Child Behavior Checklist (CBCL): the Chinese version of the questionnaire was completed by the parents, and it covered multiple dimensions. Among these, the sum of the first three subscales constituted the Internalizing Problems Scale, while the sum of the last two subscales constituted the Externalizing Problems Scale. (2) Swanson, Nolan, and Pelham Rating Scale—IV (SNAP‐IV): It is completed by parents. The scale consists of 26 items designed to assess symptoms of attention‐deficit/hyperactivity disorder (ADHD) and oppositional defiant disorder in children. The scale is divided into three dimensions: inattention (9 items), hyperactivity/impulsivity (9 items), and oppositional defiant behavior (8 items). (3) Family background: including household income, parental education level, and family harmony. The interviews were conducted with parents one‐on‐one by qualified psychological assessors.

It should be noted that, in mainland China, early screening and diagnosis of developmental disorders such as ADHD and learning disorders (LD) are still developing. Many parents were not evaluated for such conditions during their own childhood and often lack relevant knowledge. Therefore, information regarding parental or family history of ADHD or LD was not included.

### 2.4 Statistical Analysis

All analyses were conducted using R software (version 4.5.2). The packages included tidyverse, psych, rstatix, boot, ggpubr, gghalves, and circlize, among others. Continuous variables were expressed as mean ± standard deviation (Mean ± SD), or median with interquartile range [M (P25, P75)], and categorical variables were presented as frequency (%). Normality was tested using the Shapiro‐Wilk test, and homogeneity of variances was assessed using Levene's test. Between‐group comparisons were selected based on data distribution characteristics. For normally distributed variables with equal variances, independent‐samples *t*‐tests were used. For non‐normally distributed or heteroscedastic data, Mann‐Whitney *U* tests were applied. Chi‐square tests were used for categorical data. Spearman correlation analyses were conducted depending on variable distribution. Multiple linear regression was used to explore the predictive effects of questionnaire dimensions on key cognitive task performance. To avoid multicollinearity, independent variables were screened using correlation analysis and variance inflation factor (VIF) tests before inclusion in regression models. Since the sample size was small, we applied bootstrap resampling with 5000 iterations and bias‑corrected accelerated confidence intervals (BCa CI) to improve the robustness of the statistical analysis. All tests were two‐tailed, and statistical significance was set at *p* < 0.05.

## 3 Results

### 3.1 Basic Information and Family Characteristics

A total of 65 girls were included, including 33 in the ICPP group with a median age of 8.3 (7.6, 9.7) years, and 32 in the healthy control group with a median age of 8.0 (7.0, 9.1) years. There was no significant difference in age between the two groups (*p* = 0.53). The median body mass index (BMI) was 17.65 (15.3, 19.3) kg/m^2^ in the ICPP group and 17.63 (14.6, 20.65) kg/m^2^ in the control group, with no significant between‐group difference (*p* = 0.684).

Similarly, no notable difference was observed between the two groups in family background, including parents' education level, family income, and family harmony (*p* > 0.05). Detailed results are presented in Table 1.

**TABLE 1:** Baseline sociodemographic profile of participants.

	ICPP group	Healthy control group	Χ^2^	*p*	Cramer's V	95% BCa CI^a^
Father's education level						
Associate degree or below	8 (24.2%)	8 (25.0%)	0.18	0.916	0.052	(0.000, 0.082)
Bachelor's degree	15 (45.5%)	13 (40.6%)				
Master's degree or above	10 (30.3%)	11 (34.4%)				
Mother's education level						
Associate degree or below	6 (18.2%)	5 (15.6%)	0.17	0.920	0.051	(0.002, 0.078)
Bachelor's degree	20 (60.6%)	19 (59.4%)				
Master's degree or above	7 (21.2%)	8 (25.0%)				
Family income						
< ¥200,000	9 (27.3%)	11 (34.4%)	1.28	0.528	0.14	(0.007, 0.286)
¥200,000–¥500,000	18 (54.5%)	13 (40.6%)				
> ¥500,000	6 (18.2%)	8 (25.0%)				
Family harmony						
Very harmonious	13 (39.4%)	8 (25.0%)	1.71	0.426	0.162	(0.014, 0.319)
Occasional conflicts	13 (39.4%)	17 (53.1%)				
Frequent conflicts	7 (21.2%)	7 (21.9%)				

Abbreviations: BCa CI, bias‐corrected accelerated confidence intervals; ICPP, idiopathic central precocious puberty.

^a^95% BCa CI was for the Cramer's V.

### 3.2 Comparison of EF Tasks Between Groups

For the EF tasks, the ICPP group showed significantly slower RT in the Flanker task compared with the control group [1.90s (1.34s, 2.35s) vs. 1.38s (1.19s, 1.73s), *p* = 0.005]. Bootstrap analysis confirmed the stability of this finding, with a 95% BCa CI for the median difference of (0.11, 0.91), indicating that girls with ICPP responded significantly slower on this task.

**FIGURE 5:**
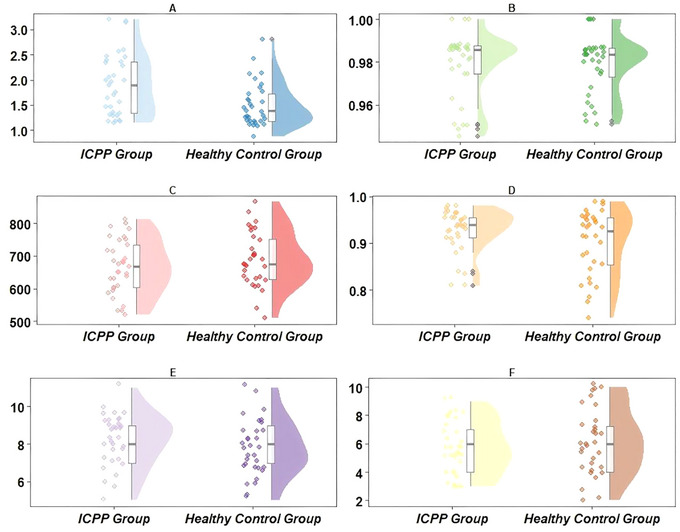
Group comparisons of EF task performance. (A) Flanker RT (s); (B) Flanker ACC; (C) Go/No‐Go RT (ms); (D) Go/No‐Go ACC; (E) Maximum forward span; (F) Maximum backward span. ACC, accuracy; RT, reaction time.

For the remaining indicators, no significant differences were observed between the two groups. This included the ACC of the Flanker task [0.99 (0.98, 0.99) vs. 0.98 (0.97, 0.989)], as well as the RT [675.75 ms (606.50 ms, 741.86 ms) vs. 675.24 ms (629.48 ms, 751.97 ms)] and ACC [0.94 (0.91, 0.96) vs. 0.93 (0.85, 0.96)] of the Go/No‐Go task. All corresponding *p* values were greater than 0.05. In the working memory assessments, the two groups showed highly similar performance. The maximum forward span was 8 (7, 9) in both groups, and the maximum backward span was 6 (4, 7) in both groups, with no significant differences (*p* > 0.05). Detailed results are presented in Figure 5.

### 3.3 Comparison of Behavioral Scale Scores

We compared the scores of the ICPP group and the healthy control group on the SNAP‐IV and the CBCL. Across the five dimensions evaluated by these two scales, the ICPP group demonstrated significantly more severe symptoms in the internalizing problems, and this trend was confirmed by bootstrap analysis. For the remaining dimensions, the ICPP group performed slightly better, but none of these differences reached statistical significance. Detailed results are presented in Table 2.

**TABLE 2:** Comparison of SNAP‐IV and CBCL scores between ICPP and control groups.

	ICPP group	Healthy control group	*z*	*p*	Median difference^a^	95% BCa CI^b^
Attention deficit	6.0 (2.0, 8.0)	7.0 (3.0, 10.0)	−0.958	0.340	−1.0	(−5.0, 3.0)
Hyperactivity impulsivity	1.0 (0.0, 3.0)	3.0 (0.75, 5.0)	−1.535	0.118	−2.0	(−5.0, −1.5)
Oppositional defiant	3.0 (1.0, 4.0)	3.0 (2.0, 7.0)	−0.623	0.534	0.0	(−4.4, 0.5)
Internalizing problems	8.0 (6.0, 13.0)	3.5 (3.0, 8.0)	3.175	0.001^**^	4.5	(1.5, 6.5)
Externalizing problems	5.0 (3.0, 8.0)	7.0 (3.75, 9.0)	−1.338	0.181	−2.0	(−5.0, 0.0)

Abbreviations: BCa CI, bias‐corrected accelerated confidence intervals; CBCL, child behavior checklist; EF, executive function; ICPP, idiopathic central precocious puberty; SNAP‐IV, Swanson, Nolan, and Pelham rating scale—IV.

^a^Median Difference = Median (ICPP Group)—Median (Healthy Control Group).

^b^95% BCa CI was for the median difference.

^*^ *p *< 0.05;^**^ *p *< 0.01. 95%.

### 3.4 Correlation Analysis of Flanker RT

As shown in the previous comparisons of EF tasks, only the RT in the Flanker task demonstrated a significant group difference. To further understand the correlational structure of Flanker RT in both the total sample and within the ICPP group, we conducted Spearman correlation analyses separately for all participants and for the ICPP group.

**FIGURE 6:**
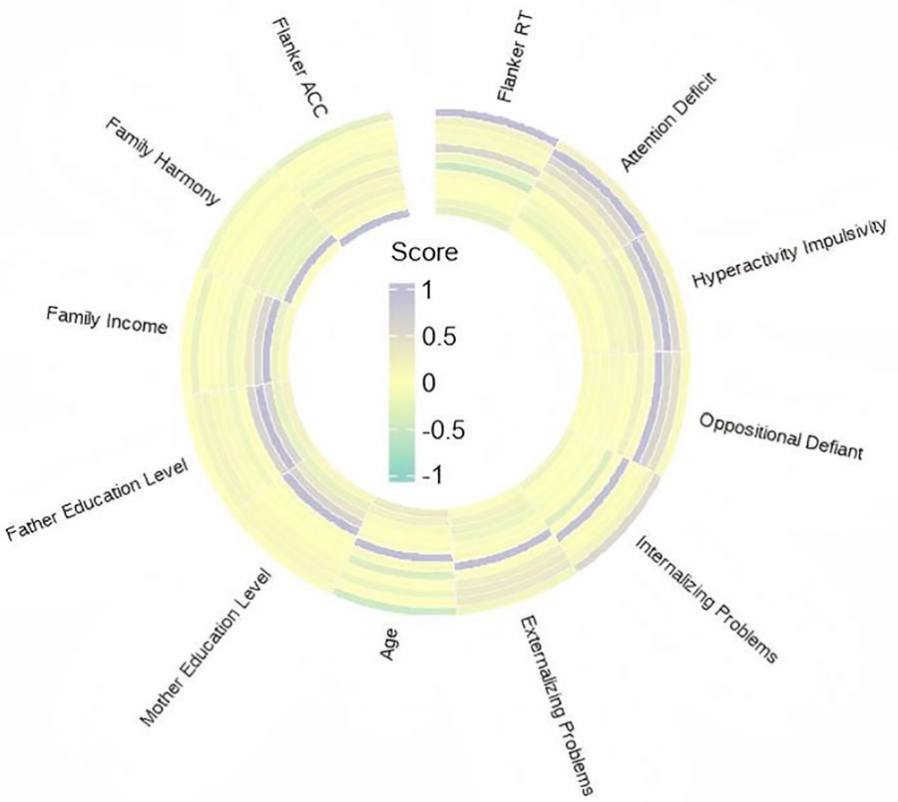
Correlation heatmap of flanker RT in the total sample. ACC, accuracy; EF, executive function; RT, reaction time.

In the total sample, Flanker RT was positively correlated with internalizing problem scores (*r* = 0.592, *p* < 0.001, BCa 95% CI: 0.374, 0.755), indicating that more severe internalizing problems were associated with longer RT. Conversely, Flanker RT was negatively correlated with age (*r* = −0.507, *p* < 0.001, BCa 95% CI: −0.667, −0.303), suggesting that older age may be related to improved EF. In addition, Flanker RT showed a negative correlation with its own ACC (*r* = −0.294, *p* = 0.017, BCa 95% CI: −0.543 to −0.006). Other variables, including attention dimensions and family background factors, were not significantly correlated with Flanker RT (all *p* > 0.05, BCa CIs containing zero). Detailed results are presented in Figure 6.

**FIGURE 7:**
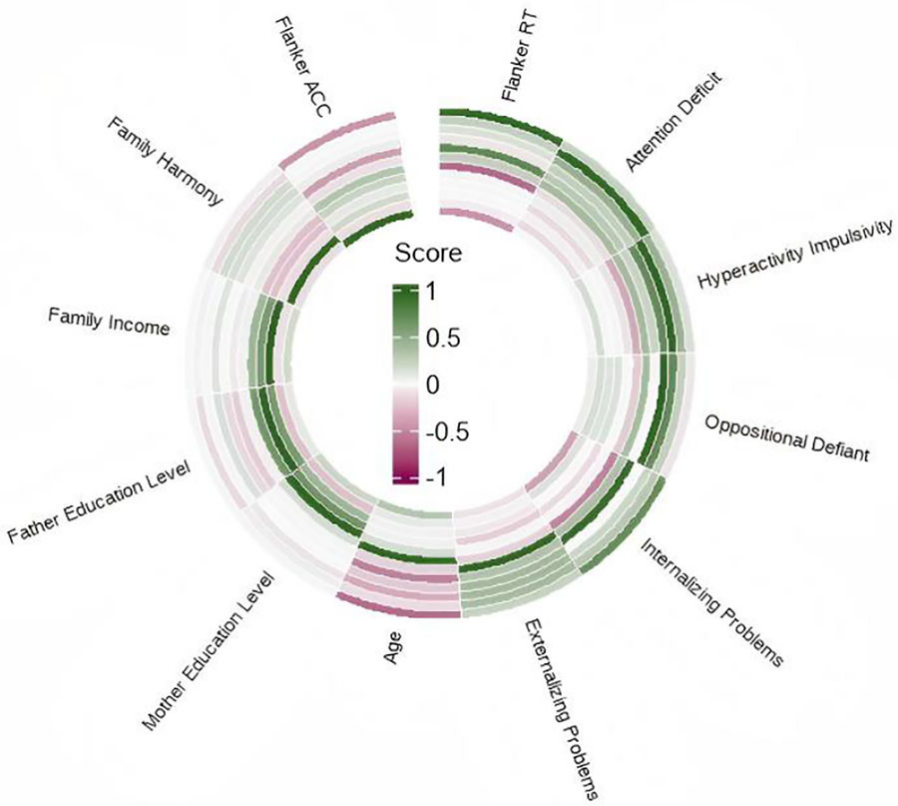
Correlation heatmap of flanker RT in the ICPP group. ACC, accuracy; ICPP, idiopathic central precocious puberty; RT, reaction time.

In the analysis of girls with ICPP, the pattern of correlations was consistent with the total sample. Flanker RT remained significantly positively correlated with internalizing problem scores (*r* = 0.766, *p* < 0.001, 95% BCa CI: 0.514 to 0.912). Similarly, Flanker RT showed a significant negative correlation with age (*r* = −0.678, *p* < 0.001, BCa 95% CI: −0.819 to −0.448). Flanker ACC also maintained a significant negative correlation with RT (*r* = −0.491, *p* = 0.003, BCa 95% CI: −0.771 to −0.115). Other variables showed no statistically significant associations with RT (all *p* > 0.05, with 95% BCa CI crossing zero). Detailed results are presented in Figure 7.

### 3.5 Multiple Regression Model for Flanker RT

To further examine the factors influencing EF in girls with ICPP, we constructed a multiple linear regression model to evaluate the independent contributions of several variables to Flanker RT. Although family income did not show a significant effect in the present study, it is an important indicator of socioeconomic status and has been consistently linked to children's cognitive performance and emotional regulation in previous literature (Ming et al. 2021). Therefore, in addition to internalizing problems and age, both of which showed significant group differences and correlations in earlier analyses, we included family income in the analysis.

In the total sample of girls, the regression model demonstrated a good performance (*R* ^2^ = 0.55, adjusted *R* ^2^ = 0.53, *p* < 0.001). Internalizing problem scores and age were both significant predictors of Flanker RT, indicating that higher levels of internalizing problems were associated with longer RT, whereas older age was associated with shorter RT. Family income did not show a significant predictive value (*p* = 0.071). All VIF values were below 5, and tolerance values were acceptable, suggesting the absence of multicollinearity. Detailed results are presented in Table 3.

**TABLE 3:** Multiple linear regression analysis for flanker RT in the total sample of girls.

	*B*	*t*	*p*	95% BCa CI^a^	VIF	Tolerance
Family income	−0.07	−1.087	0.281	(−0.216, 0.068)	1.011	0.989
Age	−0.11	−3.259	0.002^**^	(−0.177, −0.046)	1.177	0.850
Internalizing problems	0.07	6.244	<0.001^**^	(0.037, 0.093)	1.171	0.854

Abbreviations: BCa CI, bias‐corrected accelerated confidence intervals; ICPP, idiopathic central precocious puberty; RT, reaction time; VIF, variance inflation factor.

^a^95% BCa CI was for the unstandardized regression coefficient (*B*).

^*^ *p *< 0.05; ^**^ *p* < 0.01.

In the ICPP group, the regression model showed that *R*_2_ = 0.76, adjusted *R*_2_ = 0.73, *p* < 0.001. Internalizing problem scores and age remained significant predictors of Flanker RT, further supporting the notion that emotional difficulties exert an independent influence on EF within ICPP girls. Family income was not statistically significant (*p* = 0.804). Both VIF and tolerance values were within acceptable ranges, indicating no issues of multicollinearity. Detailed results are presented in Table 4.

**TABLE 4:** Multiple linear regression analysis for flanker RT in girls with ICPP.

	*B*	*t*	*p*	95% BCa CI^a^	VIF	Tolerance
Family income	−0.02	−0.257	0.799	(−0.195, 0.16)	1.017	0.983
Age	−0.10	−2.229	0.034^*^	(−0.188, −0.019)	1.558	0.642
Internalizing problems	0.09	6.077	<0.001^**^	(0.055, 0.117)	1.566	0.639

Abbreviations: BCa CI, bias‐corrected accelerated confidence intervals; ICPP, idiopathic central precocious puberty; RT, reaction time; VIF, variance inflation factor.

^a^95% BCa CI was for the unstandardized regression coefficient (*B*).

^*^ *p *< 0.05; ^**^ *p* < 0.01.

## 4 Discussion

### 4.1 Hormones in Maintaining Cognitive Stability

The HPGA not only regulates sexual maturation but also contributes to brain structure development and cognitive evolution. During puberty, heightened activity of sex hormones promotes white matter growth, which enhances the speed and efficiency of neural signal transmission. This progress is directly related to increased myelination. On the other hand, gray matter volume undergoes a decline after initial growth, a phenomenon attributed to synaptic pruning. This adaptive mechanism strengthens frequently used neural connections while eliminating those that are not commonly used (Spear 2013, Sakai 2020, Best and Ban 2021).

Furthermore, sex hormones significantly influence neurotransmitter activity within the brain, particularly involving dopamine and serotonin systems. These neurochemical changes are considered potential underlying factors for the emotional fluctuations, heightened reward sensitivity, and increased risk‐taking behavior observed during adolescence (Kuhn et al. 2010, Helmbold et al. 2015). Growing evidence indicates that hormone fluctuation within the HPGA is closely linked to EF. Both androgens and estrogens can influence attention, decision‐making, and cognitive control by modulating neural circuitry in brain regions, such as the prefrontal and mesocorticolimbic regions (Tobiansky et al. 2018, Keenan et al. 2001).

Abnormal alterations of the HPGA may lead to multiple negative cognitive outcomes. Studies in adults have shown that sex hormone dysregulation is significantly associated with cognitive impairment. For instance, postmenopausal women with low estrogen and high luteinizing hormone levels often exhibit deficits in working and spatial memory, which may contribute to the development of neurodegenerative disorders such as Alzheimer's disease (Mervosh and Devi 2025, Mey et al. 2021). Moreover, abnormal fluctuations in sex hormone levels have been linked to emotional instability and may act as triggers of affective disorders like depression and anxiety (Wium‐Andersen et al. 2022, Gordon et al. 2019).

HPGA dysfunction is not limited to adults. It can also have long‐term effects on learning and cognition in children. For instance, children with Turner syndrome often present with low sex hormone levels. Studies have shown that approximately one‐quarter of these girls suffer from ADHD (Reimann et al. 2020). Beyond attention deficit, these children may face additional cognitive difficulties, such as impaired memory and mathematical learning challenges (Hutaff‐Lee et al. 2019).

### 4.2 Impact of ICPP on EF

Early in this century, researchers proposed the developmental readiness hypothesis, suggesting that children with PP may lack the emotional and cognitive skills needed to cope with rapid physical and psychological changes, potentially leading to behavioral issues (Marceau et al. 2011, Mendle et al. 2007). Recent neuroimaging studies have provided strong biological evidence supporting this hypothesis. Chen et al. found that girls with CPP exhibited reduced gray matter volume (GMV) in the left insula and left fusiform gyrus, along with weakened functional connectivity between the insula and middle frontal gyrus, and between the fusiform gyrus and amygdala (Chen et al. 2019). Zhou et al. (2021) further confirmed that, in the reactivated state of the HPGA, GMV increased in the bilateral lingual gyri but decreased in the right inferior orbitofrontal gyrus, with the latter showing a significant correlation with follicle‐stimulating hormone levels. In addition, Yoshii et al. (2023) reported that CPP girls displayed increased cortical thickness in the right precuneus, contrasting with the gradual thinning trend observed in normal adolescent girls. These studies suggest that hormone alterations in CPP may affect the emotion‐cognition network through the HPGA‐sex hormone pathway, influencing brain regions involved in emotional processing, conflict monitoring, decision‐making, and executive control. Consequently, these changes may further impact daily functioning and academic performance in girls with ICPP.

Neuroimaging studies consistently suggest that children with ICPP may exhibit impairment in EF. However, EF is a multidimensional construct, including inhibitory control, working memory, and cognitive flexibility, among others. And different tasks may show different aspects of these processes (Miyake et al. 2000). Developmental psychology researches indicate that in early childhood these dimensions of EF are not fully differentiated, and are in an integrated stage of development. Inhibitory control and working memory mature earlier and serve as the foundation for more complex functions, such as cognitive flexibility and planning ability (Best and Miller 2010). Therefore, we focused on these two core dimensions to understand these girls’ EF while minimizing assessment burden.

In the working memory tasks, girls with ICPP performed similarly to the healthy controls, suggesting no apparent impairment in this dimension. This finding is consistent with previous studies reporting relatively stable performance in memory‐related cognitive tasks in children with early pubertal onset (Wojniusz et al. 2016). It may indicate that working memory is relatively resistant to the effects of changing sex hormones or remains stable before ICPP progresses to more complex cognitive impairments.

In contrast, significant differences were observed in inhibitory control tasks. In the Flanker task, the ICPP group showed slower RT than controls, whereas no significant group difference was observed in the Go/No‐Go task. This discrepancy may reflect the different cognitive demands of the two tasks. The Flanker task primarily measures the ability to filter competing stimuli and allocate attention resources, whereas the Go/No‐Go task focuses more on the inhibition of prepotent responses (Zhang et al. 2024). Thus, these findings suggest that inhibitory control difficulties in girls with ICPP may be more pronounced in interference control and attention allocation, rather than in impulsive response inhibition.

These behavioral differences are consistent with the findings from the behavioral scales. On the CBCL, the ICPP group scored significantly higher on internalizing problems, but no difference was found in externalizing problems. This aligns with previous evidence suggesting a close association between internalizing problems and interference control (Dell'Acqua et al. 2023). Subsequent correlation analyses and multiple linear regression conducted for the total sample and within the ICPP group confirmed a robust correlation between Flanker performance and internalizing problems. This suggests that the relationship between interference control and internalizing problems may reflect a broader neuropsychological mechanism, not limited to girls with ICPP.

Unfortunately, this study cannot determine the causal relationship between internalizing problems and interference control. However, based on previous research, we believe the association may be bidirectional. On one hand, internalizing symptoms such as anxiety or depression may intensify emotional interference, making it more difficult for individuals to detach their attention from task‐irrelevant or negative information (Eysenck et al. 2007, Koay and Van Meter 2023). On the other hand, impaired EF, particularly poor inhibitory control, may weaken the individual's ability to regulate negative emotions, potentially increasing the risk of developing internalizing problems (Booth et al. 2019). This hypothesis still needs further validation through longitudinal and experimental studies to clarify the potential mechanisms involved.

An interesting problem is that despite impaired interference control, girls with ICPP did not show abnormalities on the attention deficit dimension of the SNAP‐IV. This phenomenon may be related to cultural factors. In the Chinese cultural context, traits like being “quiet” and “obedient” are often viewed as markers of a good child. Parents tend to be more sensitive to externalizing behaviors, such as hyperactivity or aggression, while underrecognizing inattention or internal emotional distress (Luo et al. 2013). This low sensitivity to “internalizing‐type” attention problems may lead to normal SNAP‐IV scores that fail to detect underlying attention allocation difficulties.

In similar cultural contexts, especially in East Asian regions, parental reports should be treated with caution, and multiple sources should be introduced to avoid assessment bias. First, attention allocation issues are often more pronounced in long‐duration learning environments such as classrooms. Therefore, it is recommended to include teacher feedback during interviews (Li et al. 2024). Second, children's self‐evaluation can be appropriately incorporated. Research suggests that some children can recognize changes in their own emotional states, but may hesitate to share these experiences with parents due to shame or fear of being dismissed. In contrast, they may feel more comfortable expressing their feelings to clinicians, offering more direct emotional cues (Michels et al. 2013). Additionally, as in the present study, objective attention tasks can serve as an important complement to parent reports. These tasks provide indicators of attention control that are independent of subjective perceptions.

### 4.3 Cognitive Effects of GnRHa Remain Uncertain in ICPP

Given that abnormal sex hormones may affect cognitive function, it is naturally assumed that GnRHa, as a first‐line treatment, will help improve cognitive function. However, studies have suggested that GnRHa treatment may further impair cognitive function. In rodent and sheep models, researchers found that GnRHa treatment after puberty could negatively impact behavior, stress regulation, and spatial memory (Anacker et al. 2021, Hough et al. 2017). A clinical study also found that girls with CPP who received GnRHa had slightly lower IQ scores than controls (Wojniusz et al. 2016). Although the difference was not statistically significant, the finding has raised concerns among experts (Hayes 2017).

Therefore, clinical evaluation of ICPP should not be limited to growth rate, bone age, and hormone levels. Patients need more comprehensive assessment. Cognitive function and emotional status are necessary to make cautious treatment decisions. For girls who have already started GnRHa therapy, it is recommended to monitor EF, emotional changes, and learning performance during the treatment. If signs of cognitive decline were found, timely and appropriate interventions should be introduced to reduce potential risks.

### 4.4 Limitations and Perspectives

We acknowledge that this study has limitations. (1) One limitation of this study is the absence of a standardized intelligence assessment, such as the Wechsler Intelligence Scale for Children (WISC), which prevents the complete exclusion of the potential influence of generalized cognitive abilities on task performance. Although the two groups were comparable in age and educational background, and the differences were primarily reflected in Flanker RT rather than multiple cognitive domains, we still cannot rule out the possible effect of intelligence. Future studies should consider incorporating formal intelligence measures to better delineate the independent contribution of EF. (2) We used a cross‐sectional design with a relatively limited sample size, and the follow‐up data have not yet been obtained. (3) Another limitation of this study is that we relied primarily on parent‐reported measures and did not include teacher reports or child self‐reports. Given that attention and emotional difficulties may manifest differently across settings, the absence of multi‐informant data may limit the comprehensiveness of our assessment.

## 5 Conclusion

Our study found that girls with ICPP showed weaker interference control, and this impairment was significantly associated with internalizing problem scores. These findings suggest that EF impairment in ICPP is not generalized but is characterized by reduced capacity for attention allocation and information filtering, and it may be modulated by emotional states. These results highlight the need for pediatricians to pay more attention to the interaction between emotion and cognition in children with ICPP.

## Author Contributions

Conceptualization: Qian Zhang and Nai‐jun Wan. Study design: Qian Zhang and Nai‐jun Wan. Data acquisition: Qian Zhang. Writing – original draft preparation: Qian Zhang. Writing – review and editing: Qian Zhang and Nai‐jun Wan.

## Funding

Project supported by Beijing JST Research Funding (code: QN202422), which provides financial support in the design of the study and collection, analysis, and interpretation of data, and in writing the manuscript.

## Disclosure

AI: The authors used AI‐assisted tools exclusively to enhance language, grammar and readability. No generative AI was employed in study design, data collection, analysis, interpretation or the writing of scientific content.

## Ethics Statement

Ethical approval for this study was obtained from the Beijing Jishuitan Hospital, Capital Medical University, Beijing, China (approval number: K2024‐195‐00). All data collection was conducted with written informed consent from parents or legal guardians. Additionally, for children aged 8 years or older, assent from the child was also obtained. All data were handled in compliance with applicable data protection regulations, and confidentiality was strictly maintained throughout the study. This study complies with the ethical principles outlined in the Declaration of Helsinki.

## Conflicts of Interest

The authors declare that they have no competing interests.

## Data Availability

The datasets analyzed during the current study are available from the corresponding author on reasonable request.
